# Effect of Paricalcitol vs Calcitriol on Hemoglobin Levels in Chronic Kidney Disease Patients: A Randomized Trial

**DOI:** 10.1371/journal.pone.0118174

**Published:** 2015-03-17

**Authors:** Eleonora Riccio, Massimo Sabbatini, Dario Bruzzese, Ivana Capuano, Silvia Migliaccio, Michele Andreucci, Antonio Pisani

**Affiliations:** 1 Chair of Nephrology, Department of Public Health, University Federico II of Naples, Naples, Italy; 2 Chair of Statistics, Department of Public Health, University Federico II of Naples, Naples, Italy; 3 Unit of Nephrology, University Magna Graecia, Catanzaro, Italy; University of Ottawa, CANADA

## Abstract

**Background:**

Recent studies suggest that vitamin D deficiency represents an additional cofactor of renal anemia, with several mechanisms accounting for this relationship. In line with it, the administration of vitamin D or its analogues has been associated with an improvement of anemia. There are no data, however, about a direct effect of paricalcitol on hemoglobin (Hb) levels. Therefore, we conducted a study to determine whether paricalcitol, compared to calcitriol, improves anemia in patients with chronic kidney disease (CKD).

**Methods:**

In this randomized trial 60 CKD patients stage 3b-5 and anemia (Hb levels: 10-12.5 g/dL) were assigned (1:1) to receive low doses of calcitriol (Group Calcitriol) or paricalcitol (Group Paricalcitol) for 6 months. All the patients had normal values of plasma calcium, phosphorus and PTH, a stable iron balance, and normal values of C-Reactive Protein. The primary endpoint was to evaluate the effects of the two treatments on Hb levels; the modifications in 24hr-proteinuria (UProt) were also evaluated.

**Results:**

A significant Group x Time interaction effect was observed in the longitudinal analysis of Hb levels (F(1,172)=31.4, p<0.001). Subjects in Paricalcitol experienced a significant monthly increase of Hb levels equal to +0.16 g/dL [95% C.I. 0.10 to +0.22, p<0.001) while in Group Calcitriol, Hb decrease throughout the follow-up with an average monthly rate of -0.10 g/dL (95% C.I.: -0.17 to -0.04, p<0.001). In Group Paricalcitol, UProt was significantly reduced after 6 months [0.35 (0.1-1.2) vs 0.59 (0.2-1.6), p<0.01], whereas no significant difference emerged in Group Calcitriol. Plasma levels of calcium, phosphate, PTH and of inflammation markers remained in the normal range in both groups throughout the study.

**Conclusions:**

Short-term exposure to paricalcitol results in an independent increase in Hb levels, which occurred with no modification of iron balance, inflammatory markers, and PTH plasma concentrations, and was associated with a decrease in UProt.

**Trial Registration:**

ClinicalTrials.gov NCT01768351

## Introduction

Recent literature strongly emphasizes the pleiotropic effects of vitamin D independent of calcium and phosphate homeostasis [1], which include reduced atherogenesis, lower renin-angiotensin-system activity, reduced proteinuria, decreased inflammation, and improved endothelial function [2–4]. Nevertheless, vitamin D deficiency or insufficiency is prevalent all over the world in each population segment and is considered a real pandemic disease [5].

Vitamin D also affects the haemopoietic system; its receptors (VDRs), in fact, are present in bone marrow, including stromal and accessory cells [6] and vitamin D deficiency represents an additional cofactor of anemia, beyond erythropoietin (EPO) and iron deficiency or inflammation [7, 8]; significant relationships, moreover, exist between plasma levels of 25-hydroxyvitamin D and hemoglobin (Hb) concentrations in health [9] and disease [8, 10].

Several mechanisms may account for this association: “in vitro” studies on red cells precursors of bone marrow demonstrate that calcitriol directly increases EPO-receptor expression and synergistically stimulates cell proliferation along with EPO [11, 12]. In addition, vitamin D shows anti-inflammatory properties that improve responsiveness to EPO through the reduced production of hepcidin and pro-inflammatory cytokines [7, 13], whose concentrations increase during vitamin D deficiency in bone marrow and impair erythropoiesis [14]; VDRs activation, conversely, up-regulates the lymphocytic release of anti-inflammatory interleukin-10 (IL-10) which exerts proliferative effects on erythroid progenitors [12, 15].

Finally, in patients with chronic kidney disease (CKD) the positive effects of vitamin D on erythropoiesis could also be related to its suppressive effects on PTH, which directly inhibits erythroid progenitors, EPO synthesis, and red blood cells survival [16]. Recent reports, however, suggest that EPO resistance to high PTH levels is probably related to vitamin D deficiency, whose levels were not assessed in previous studies [12].

In line with these observations, the administration of vitamin D and of its analogues has been associated with an improvement of anemia and/or a reduction in EPO requirements. Alfacalcidol [17], cholecalciferol, and ergocalciferol showed a positive impact on anemia in hemodialysis (HD) patients [18], while calcitriol improved hemoglobin Hb levels and reduced the need for EPO in CKD patients on conservative management and on dialysis [12, 19]; finally, a recent study evaluating 1-year treatment with paricalcitol in 12 HD patients previously treated with calcitriol, showed an amelioration of erythropoiesis during paricalcitol treatment [20]. It is noteworthy that most of the studies evaluating the association of vitamin D deficiency with renal anemia emphasize the role of inflammation and of PTH as the main mediators of these changes [14].

To date, no randomized clinical trial (RCT) has evaluated the effects of oral paricalcitol compared to calcitriol on Hb levels, as main outcome measure in CKD patients. Therefore, we conducted a pilot RCT to determine whether the use of paricalcitol improves anemia in CKD patients. To ascertain a direct and independent effect of paricalcitol on erythroid cells, the study was carried out in patients with normal levels of PTH and devoid of overt clinical inflammation, two confounding factors in pathogenesis of renal-anemia.

## Patients and Methods

The protocol for this trial (as approved by our local ethic committee) and supporting CONSORT checklist are available as supporting information; see S1 CONSORT Checklist and S1 Protocol.

### Patients

This prospective study was conducted in the CKD Clinic of the University Federico II of Naples, Italy, where 185 consecutive patients (stage 3b-5) were screened from October 2010 to October 2012.

Inclusion criteria for the study were: age >18 years, estimated GFR (eGFR, MDRD equation) ≤ 45 ml/min/1,73 m^2^, Hb levels between 10 and 12.5 g/dL, normal transferrin saturation (TSAT, 20–40%), plasma ferritin levels ≥100 ng/mL, normal mean corpuscular volume (MCV, 85–95 fL), parathormone (PTH) serum levels between 20 and 300 pg/mL, according to the suggested values for kidney disease stage, and calcium and phosphate plasma levels within their normal values (i.e. <10.5 mg/dl, and <4.5 mg/dl, respectively), controlled by a low protein diet (0.7–0.9 g/kg b.w./day), calcium supplements and phosphate binders.

Exclusion criteria were: presence of inflammatory, infectious disease or surgical interventions in the last 3 months, high-sensitivity C-reactive protein (CRP) levels >3 mg/dl (normal values: 0–5 mg/ml), hematological disorders or bleeding in the last 6 months, malignancies, treatment with immunosuppressive drugs, poorly controlled hypertension (>170/100 mmHg), presence of clinical/ECG signs of cardiovascular disease in the last 3 months.

Withdrawal from the study was considered in case of malnutrition (loss of body weight >5% in 1 month or BMI < 20 kg/m^2^ with serum albumin levels <3.2 g/dl), need to start dialysis (eGFR ≤6 ml/min, K^+^>6.0 mEq/L, intractable hypertension) or Hb values falling below 10 g/dl. Pharmacological and non-pharmacological therapies were prescribed to each patient to achieve the therapeutic targets in keeping with K/DOQI CKD guidelines for stages 3b-5. Patients previously treated with vitamin D or its analogues discontinued such therapy at least 1 month before starting the study. Informed written consent was obtained from each patient. The trial was approved in September 2010 by our local Medic Ethics Committee (Ethic Commettee “Carlo Romano” of Federico II University of Naples) and was in adherence with the Declaration of Helsinki. It was registered in ClinicalTrials.gov with Identifier: NCT01768351. The authors confirm that all ongoing and related trials for this drug/intervention are registered.

### Study design and procedures

**Fig 1:**
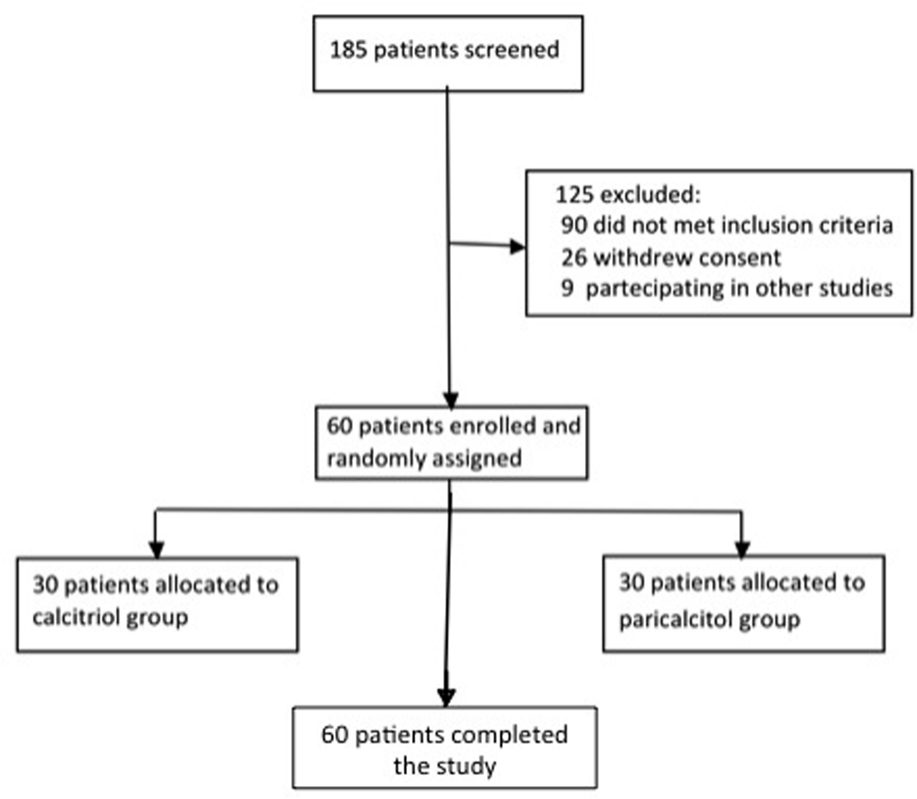
Flow chart of patient selection.

This was a prospective RCT in which the randomization list was generated by a computer and kept concealed in numbered, sealed envelopes opened in sequence by administrative staff personnel. According to our criteria, 60 patients were enrolled in the study and were randomly assigned (1:1) to receive treatment with fixed low doses of calcitriol (Group Calcitriol) or paricalcitol (Group Paricalcitol), irrespective of baseline PTH levels (Fig. 1). The treatment phase lasted 6 months and subjects were dosed in a 4:1 ratio of paricalcitol (1 mcg/day) to calcitriol (0.5 mcg/every other day), based on previous clinical studies that compared the two vitamin D compounds.

All the patients were examined at baseline (T0) and after two (T2), four (T4) and six months (T6) of treatment, in which body weight and sitting blood pressure (BP, average of 3 measurements in 15 min) were evaluated and blood was withdrawn to determine the main laboratory data. Urinary protein excretion was also evaluated in 24-hour samples (UProt). At T0 and T6, plasma levels of vitamin B_12_ and folate were also measured in all the patients.

Standard laboratory procedures were used for blood and urinary measurements.

All the patients were maintained at the same dietary regimen and the same pharmacological therapies throughout the study, including phosphate binders, angiotensin converting enzyme inhibitors (ACE-I), angiotensin receptor blockers (ARBs), and erythropoiesis-stimulating agents (ESA); if Hb values resulted >13 g/dl, however, ESA dosage was reduced by 25%; similarly, oral iron therapy was introduced (oral sulfate iron, 105 mg/day) if TSAT fell below 15%. No further calcium supplement was administered to any patient during the follow-up.

### End-points

The primary endpoint of the study was to evaluate the effects of low doses of two different treatments, namely calcitriol and paricalcitol, on Hb plasma levels during a follow-up period of 6 months in CKD patients with stable levels of calcium, phosphorus and PTH. As secondary end point, the modifications in UProt with both drugs were also evaluated.

### Statistical analysis

Data with normal distribution were summarized as means ± standard deviation and those with non-normal distribution as medians and inter-quartile range. Between groups comparisons of independent variables were performed by Student’s t-test and Mann–Whitney U-test for normally and not-normally distributed variables, respectively. Within patients comparisons were performed by paired t-Test or Wilcoxon Rank Test, as appropriate. Differences of categorical variables between two groups were investigated by the χ^2^ test. A p value <0.05 was considered statistically significant.

The independent relationship between the treatments (paricalcitol versus calcitriol) and hemoglobin data over time (i.e. hemoglobin values at T2, T4 and T6, dependent variable) was investigated by a multiple linear mixed model (LMM) with group, time and group x time interaction as main predictors and by adjusting for hemoglobin value at baseline and other potential confounders (i.e. the variables resulting different between the two Groups at baseline with P<0.10 at comparative analysis). Both time (expressed in months from baseline) and subject were considered as random effect and an unstructured variance-covariance structure was applied to account for within-patient dependence of hemoglobin values over time. In multiple LMM analysis data were expressed as regression coefficient, 95% confidence interval and P value. Data were analyzed using the Statistical Package for Social Sciences (SPSS) for Windows, version 20.0 software (SPSS Inc., IL, USA).

## Results

### Baseline data (Tables, 1,2)

At baseline, the two groups were comparable for age, body weight, eGFR, and use of ESA, phosphate binders, calcium, iron supplements and vitamin D analogs. Significant differences, conversely, were detected in number of females, greater in Paricalcitol Group, in number of diabetic patients and in use of ACE-I, both higher in Calcitriol Group. The use of antihypertensive therapies was similar between the groups (data not shown).

**Table 1:** Demographic characteristics, diagnosis of renal disease and drug treatments in the 2 groups under study (n = 30 each).

	ALL	Paricalcitol	Calcitriol
Age (years)	56.8±16.8	59.9±15.1	54.7±18.3
Female gender	20 (33%)	15 (50%)	5 (17%)°
Body weight (kg)	74.4±13.7	73.3±13.6	75.4±13.8
eGFR (ml/min/1.73m^2^)	26.4±9.9	25.4±9.6	27.4±10.3
CKD stage (3b/4/5/)	19/39/2	9/20/1	10/19/1
GN	15 (25%)	7 (23%)	8 (27%)
DM	12 (20%)	3 (10%)	9 (30%)°
ADPKD	8 (13%)	3 (10%)	5 (17%)
Urologic causes	2 (2%)	2 (7%)	0 (0%)
Other/unknown	23 (30%)	15 (50%)	8 (27%)
ACE-I	32 (53%)	12 (40%)	20 (67%)°
ARB	27 (45%)	16 (53%)	11 (37%)
Calcium suppl.	11 (18%)	6 (20%)	5 (17%)
Phosphate binders	15 (25%)	7 (23%)	8 (27%)
Iron	13 (22%)	7 (23%)	6 (20%)
ESA	8 (13%)	4 (13%)	4 (13%)
Vitamin D analogs	15 (25%)	8 (27%)	7 (23%)

Data are expressed as means ± SD; data in brackets express the % values in that group.

° p<0.05 between groups

Abbreviations: eGFR, estimated glomerular filtration rate; CKD, chronic kidney disease; GN, glomerulonephritis; DM: diabetes mellitus; ADPKD, adult polycystic kidney disease; ACE-I, inhibitors of angiotensin converting enzyme; ARB, angiotensin receptor blockers; ESA, erythropoiesis stimulating agents.

Primary kidney disease was classified according to the European Renal Association codes.

**Table 2:** Main laboratory data of the patients under study (ALL, N = 60), and in the two groups after randomization (n = 30 in both groups).

	ALL	Paricalcitol	Calcitriol
Hb (g/dL)	11.8±0.7	11.7±0.8	12.0±0.7
Ferritin (ng/mL)	190.5±116.9	175.2±118.2	205.7±116.1
TSAT (%)	28.1±7.4	27.4±7.6	28.8±7.4
Vit. B12 (pg/mL)	514.7±405.4	432.0±174.4	595.4±138.6
Folate (ng/mL)	6.6 (4.7–9.1)	6.8 (4.8–8.8)	6.3 (4.7–9.8)
Calcium (mg/dL)	9.4±0.4	9.4±0.3	9.3±0.4
Phosphate (mg/dL)	3.6±0.7	3.6±0.5	3.6±0.9
PTH (pg/mL)	116.5 (92–149)	116 (44–146)	114 (38–137)
Albumin (g/dL)	4.3±0.3	4.3±0.3	4.3±0.4
hsPCR (mg/L)	1.20 (0.90–1.70)	1.20 (0.80–1.70)	1.30 (0.90–1.85)
UProt (g/24 h)	0.52 (0.18–1.47)	0.59 (0.23–1.56)	0.44 (0.14–1.35)

Data are expressed as means ± standard deviation or as median and interquartile range.

Patients showed an excellent metabolic control, and no difference was detected between the two groups in any laboratory data, including Vitamin B_12_ and folate concentrations, all in the normal range.

### Follow-up data (Table 3)

None of the 60 participating patients dropped out of the study throughout the follow-up period.

Low doses of Paricalcitol and Calcitriol did not alter the PTH-calcium-phosphate axis. In fact, no change was observed in any of these parameters throughout the study, despite a slight decrease of PTH in both Groups (NS), as expected.

**Table 3:** Main clinical and laboratory data in the two Groups under study throughout the follow-up period (n = 30 in both groups).

	Paracalcitol				Calcitriol			
	T0	T2	T4	T6	T0	T2	T4	T6
Hb	11.7±0.8	11.9±0.9*	12.2±0.9* ^#^	12.6±0.9* ^#^	12.0±0.6	11.8±0.5*	11.6±0.5*	11.4±0.5*
GFR	25.4±9.6	25.5±9.9	23.7±8.4*	23.0±8.4*	27.4±10.3	27.0±11.4	26.7±10.9	25.4±11.8
Ferritin	175.2±68.2	162.9±60.8	152.3±48.5	155.8±53.1	185.8±53.0	193.3±84.9	189.2±79.8	180.4±72.2
TSAT	27.4±7.2	27.1±6.2	27.0±6.4	25.6±5.3	25.8±7.3	28.3±8.2	27.1±5.6	25.3±5.2
Ca	9.4±0.3	9.4±0.4	9.4±0.4	9.5±0.4	9.3±0.4	9.3±0.4	9.4±0.4	9.4±0.3
P	3.6±0.5	3.7±0.5	3.8±0.5*	3.7±0.6	3.7±0.6	3.8±0.7	3.6±0.6	3.8±0.6
PTH	116 (44–146)	110 (40–102)	108 (33–101)	103 (27–98)	114 (38–137)	112 (39–121)	108 (21–118)	104 (34–120)
hsCRP	1.2 (0.8–1.7)	1.2 (0.9–2.0)	1.2 (0.8–1.7)	1.0 (0.8–1.5)	1.3 (0.9–1.9)	1.0 (0.9–2.0)	0.9 (0.8–1.5)	1.0 (0.8–1.9)
UProt	0.59 (0.2–1.6)	0.74 (0.2–1.4)	0.50 (0.1–1.3)^#^	0.35 (0.1–1.2)* ^#^	0.44 (0.1–1.3)	0.55 (0.1–1.9)	0.66 (0.0–1.8)	0.79 (0.3–1.8)
SBP	130.5±18.1	131.5±18.5	130.8±17.9	129.5±15.4	130.8±14.5	130.3±16.0	133.0±17.5	132.3±18.6
DBP	76.3±8.7	75.3±9.7	76.5±7.2	73.5±8.8	77.0±8.3	75.8±9.6	76.3±11.0	76.7±11.6

Data are expressed as means ± SD or as median and interquartile range.

* = p<0.05, minimum value, vs baseline T0;

# = p<0.05, minimum value, difference between groups (same period).

Abbreviations and measure units: Hb, Hemoglobin (g/dl); GFR, glomerular filtration rate (ml/min); Ferritin (ng/dl); TSAT, transferrin saturation (%); Ca, calcium plasma levels (mg/dl); P, phosphate plasma levels (mg/dl); PTH, parathormon (ng/ml); hsCRP, high sensitivity C reactive protein (mg/dl); UProt, 24 hours urinary protein excretion (g/day); SBP and DBP, systolic and diastolic blood pressure, respectively (mm Hg).

T0: baseline; T2, T4, and T6: 2, 4, and 6 months of follow-up.

**Fig 2:**
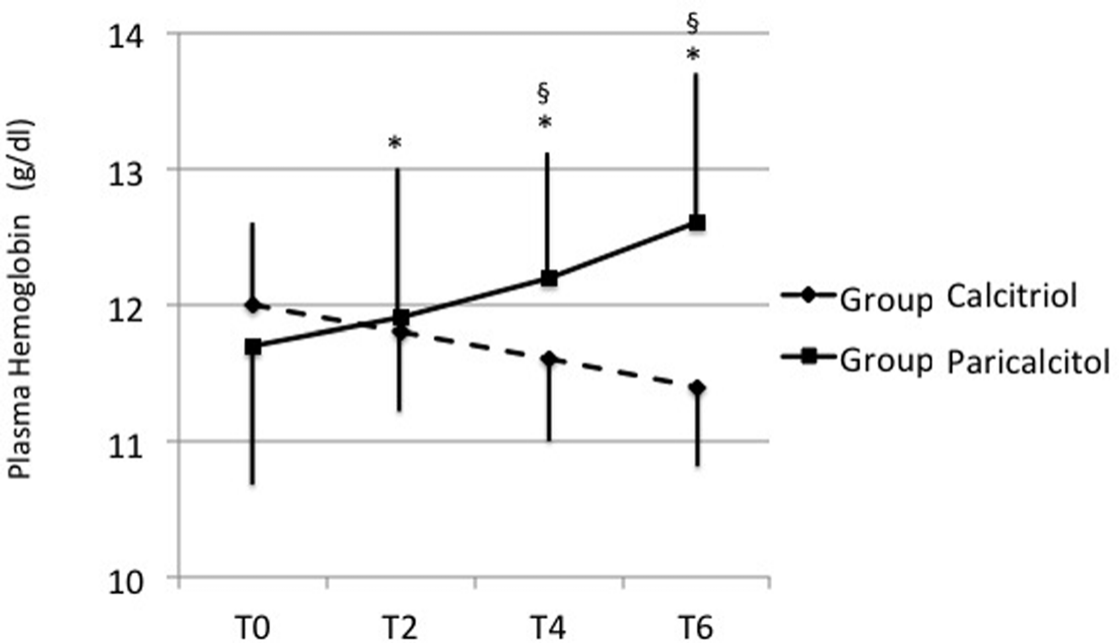
Effects of calcitriol (Group Calcitriol, dashed lines) and paricalcitol (Group Paricalcitol, solid lines) on hemoglobin plasma levels throughout the study. Data are expressed as means ± SD. Abbreviations: T0, baseline levels; T2, T4 and T6, values after 2, 4 and 6 months of follow-up with both drugs. * = p<0.05 (minimum value) vs T0; § = p<0.05 (minimum value) vs respective period of Group CALC

Hb plasma concentrations, conversely, showed a divergent pattern (Fig. 2). Patients of Group Paracalcitol showed a significant rise in Hb levels in T2 compared to baseline (+0.26 g/dL, 95% C.I. [+0.07 to +0.45], p = 0.008), which progressively increased at T6 (+0.89 g/dL, 95% C.I. [+0.64 to 1.15], p<0.001). In Group Calcitriol, conversely, a slight decline in Hb concentration was observed throughout the follow-up period leading ad T6 to a significant 5% relative mean reduction with respect to baseline (-0.61 g/dL 95% C.I. [-0.18 to -0.42], p<0.001); as a consequence, the differences in Hb levels between Paricalcitol and Calcitriol group became statistically significant at T2 (+0.57 g/dL, 95% C.I. +0.18 to +0.96], p<0.001) and increased in T4 (+1.17 g/dL, 95% C.I. [+0.73 to +1.61], p<0.001). In multiple linear mixed model, after adjusting for Hb levels at baseline and other confounding factors (gender, diabetes, and use of ACEi) a significant Treatment x Time interaction was observed in the longitudinal course of Hb levels (F(1,172) = 31.4, p<0.001). In particular the monthly average increase in Hb levels for Paricalcitol group was +0.16 g/dL [95% C.I. 0.10 to +0.22, p<0.001) while subjects in Calcitriol group experienced a significant reduction in Hb levels equal to -0.10 g/dL per month (95% C.I.: -0.17 to -0.04, p<0.001).

These changes occurred with stable ferritin plasma levels and TSAT values in both groups. Indeed, a decrease in TSAT was observed at T6 vs T0 in Calcitriol Group (28.77% at T0 vs 25.33% at T6; mean difference-3.43%, 95% C.I. [-0.53 to -6.34], p = 0.022), which, however, remained in the normal range in all the patients; accordingly, no further iron supplement was administered during the follow-up.

**Fig 3:**
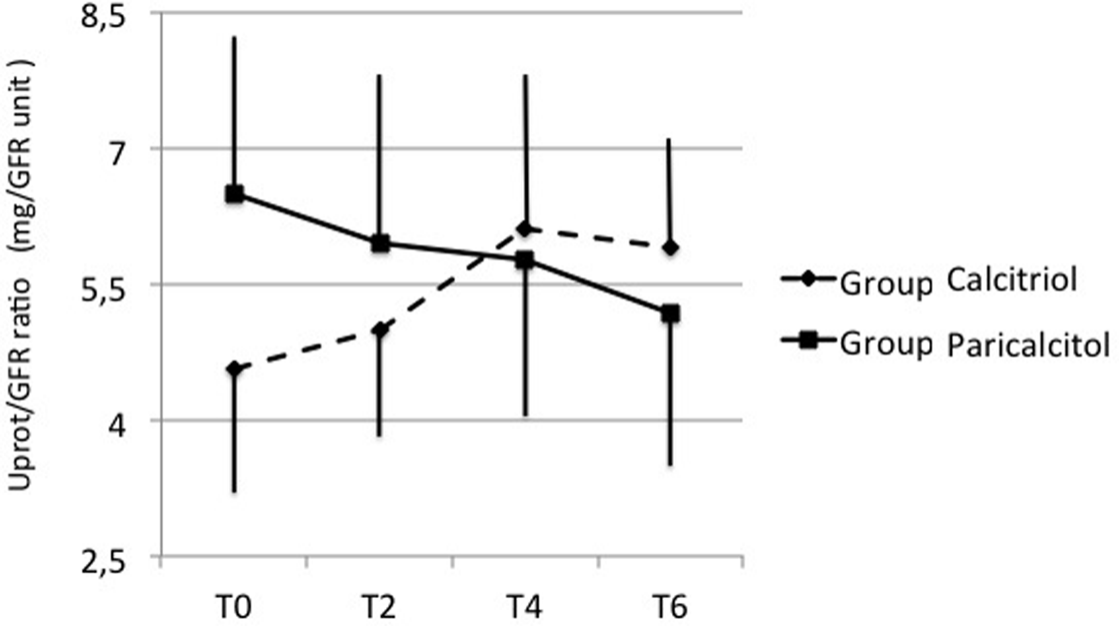
Effects of calcitriol (Group Calcitriol, dashed lines) and paricalcitol (Group Paricalcitol, solid lines) on urinary protein excretion/GFR ratio throughout the study. Data are expressed as means ± SE. Abbreviations: T0, baseline levels; T2, T4 and T6, values after 2, 4 and 6 months of follow-up with both drugs.

In Paricalcitol Group, 24-hour UProt was significantly reduced after 6 months of treatment (0.59 [0.23; 1.56] g/24h at T0 vs 0.35 [0.14; 1.16] g/24h at T6, p<0.001), whereas patients under Calcitriol exhibited numerically higher values of UProt throughout the follow-up period (+49%, T6 vs T0, NS). These modifications occurred despite concomitant decreases of eGFR in both Groups (-2.3 ml/min/1.73m^2^, 95% C.I.: -3.9 to—0.8, p = 0.003 in Group Paricalcitol and—2.0 ml/min/1.73m^2^, 95% C.I.: -3.5 to -0.5, p = 0.010 in Group Calcitriol, respectively, vs their T0); it is noteworthy that the UProt/eGFR ratio tended to decrease in Group Paricalcitol, but increased to a steady level in Group Calcitriol at T6 when compared to T0 (Fig. 3).

In both Groups hsCRP plasma concentrations did not vary and BP was well controlled during the whole study period.

According to the protocol, patients were maintained at the same pharmacological therapies throughout the follow-up period. Only one patient of Group Paricalcitol needed to reduce ESA dosage, and two patients stopped ESA treatment (one in each group).

## Discussion

The key finding of this RCT is that short-term treatment with paricalcitol results in an independent increase in Hb levels associated with a decreased urinary protein excretion, which occurred with no modification in iron balance, inflammatory markers, and PTH plasma concentrations. Despite similar clinical conditions, patients treated with calcitriol showed no benefit in Hb levels, nor in UProt.

To date, therapies with activated vitamin D are only approved for treating secondary hyperparathyroidism in CKD; a large body of experimental and clinical data, however, suggests that vitamin D and its analogues positively affect several factors enhancing the progression of CKD, like anemia, urinary protein excretion, activation of renin-angiotensin system and inflammation [21–25]. Despite some studies have suggested greater clinical benefits with paricalcitol than with calcitriol, no study has directly compared the two drugs.

Hence, our work hypothesis was to evaluate in a randomized trial the effects of paricalcitol versus calcitriol on Hb levels in patients with moderate-severe CKD. Our effort was to enroll patients with normal concentrations of calcium, phosphate and PTH (according to their CKD stage) and no clinical or laboratory sign of inflammation, since in most clinical studies the beneficial effects of vitamin D on Hb levels were observed in patients with CKD with sustained levels of inflammation and elevated PTH values, considered as important cofactors of anemia.

The present study confute this view, showing that the beneficial effect of paricalcitol on Hb plasma levels occurred independently of any change in calcium-phosphate-PTH axis, in presence of normal concentrations of inflammatory markers and with no modification in iron balance or ESA doses. And, indeed, statistics clearly demonstrates that paricalcitol represents an independent effector of Hb changes, excluding any role for confounding factors like ACE-I use and diabetes, whose prevalence was greater in Calcitriol Group. Our study indirectly confirms recent data by Patel et al. showing that the decline in Hb concentrations in CKD patients with low levels of both 25- and 1,25-vitamin D resulted fully independent of the inflammatory status and of PTH concentrations [8].

It is noteworthy that the amelioration of Hb concentration occurred in presence of unexpectedly declining values of GFR during the follow-up. Different studies, however, have suggested that paricalcitol may spuriously enhance the production of creatinine, negatively influencing the calculation of eGFR. Agarwal et al. have recently shown that creatinine production increased after one week of oral paricalcitol, but returned to baseline levels after its discontinuation [26]. Alborzi et al. have also demonstrated in patients with stage 3 CKD randomly assigned to different doses of paricalcitol that iothalamate clearance did not vary at any dosage after 1-month treatment [24]. Comparable findings have been described with oral calcitriol, which depressed creatinine clearance, but did not affect inulin clearance [27, 28]. Therefore, in both groups the observed decline in GFR could only be apparent; accordingly, the slight decline in Hb values observed in Calcitriol patients could be a consequence of the higher prevalence of diabetes or the greater use of ACE-I in this group, even if these factors did not affect the statistical analysis.

The second interesting finding was the significant decrease in urinary protein excretion observed in patients under paricalcitol. Despite the large variability of data and the limited power of our study to detect a true effect on proteinuria, the changes between the two groups after exposure to the drugs were statistically significant, with patients of Calcitriol Group exhibiting steady values of proteinuria and those under paricalcitol showing a reduced protein excretion; indeed, the UProt/GFR ratio showed a clear tendency to decline in patients of Paricalcitol Group, but remained constant in calcitriol patients, despite the greater use of ACE-I in this latter group.

The anti-proteinuric effect of paricalcitol has already been evidenced in both experimental [29, 30] and clinical studies [26, 31], and is probably mediated by a direct suppression of RAS promoter gene by paricalcitol [32], since it seems to be additive to the antiproteinuric effects of “peripheral” RAS inhibitors [21], and is not accounted for by blood pressure reduction during treatment. In our patients, in fact, BP remained constant throughout the follow-up period in both arms of the study.

A role for the reduced inflammation after paricalcitol in determining our results can be reasonably excluded, since our patients started from CRP values well below the highest normal value and can conceivably be considered not inflamed. We did not measure more accurate markers of inflammation, but several studies have clearly demonstrated that CRP levels positively correlate with the severity of anemia and EPO resistance in CKD patients, and that CRP concentrations closely reflect Interleukin-6 levels [33–36]. Alborzi et al. found a significant decrease in CRP (-50%) in 16 patients receiving paricalcitol for 1 month; these patients, however, started from CRP values 3–4 fold higher than ours and CRP levels still exceeded normal values at end of follow-up [24]. Therefore, the anti-inflammatory effects of paricalcitol in our patients seem negligible. It should be noted, however, that different studies have not confirmed the anti-inflammatory effects of Vitamin D derivatives [32, 37], questioning whether vitamin D supplementation may be considered an actual strategy to correct inflammation in CKD patients. Our results overcome these doubts, showing the independent effect of paricalcitol on Hb levels.

The present study has some limits. First, patients of both groups were highly selected; this selection, however, was necessary to minimize potential determinants of renal anemia, but it seems obvious that the beneficial effects of paricalcitol are extensible to all CKD patients. Second, we did not measure basal levels of vitamin D; Alborzi et al., however, showed that paricalcitol administration does not influence even low vitamin D concentrations, indirectly suggesting that the results after paricalcitol are independent from vitamin D plasma levels [24]. Third, the study lacks a placebo group, which could have better clarified the exact role of calcitriol in GFR reduction and urinary protein excretion. Last, it should be kept in mind that our study evaluates a laboratory outcome, which represents a surrogate of a clinical outcome; to study “hard” clinical outcomes requires longer trials and a greater number of patients.

The strength of the study, conversely, reside in the optimal clinical and metabolic control of our CKD patients, which was carefully maintained throughout the study, the first randomized study which compared the extra-calcium homeostasis effects of calcitriol and paricalcitol.

In conclusion, our study shows for the first time that paricalcitol, compared to calcitriol, exerts a beneficial effect on Hb levels in patients with advanced CKD independent of several confounding factors involved in anemia pathogenesis, like inflammation and PTH levels. Our data suggest a potential adjunctive role for this substance in the overall management of patients with CKD, mostly considering the stability of calcium, phosphate and PTH levels during a low-dose treatment.

## Supporting Information

S1 CONSORT Checklist Copy of the Consort checklist. (DOC)

S1 Protocol Copy of the trial protocol as approved by the ethics committee. (DOCX)
