# Supplementary material for: Effect of Paricalcitol vs Calcitriol on Hemoglobin Levels in Chronic Kidney Disease Patients: A Randomized Trial
Source: PLoS One. 2015 Mar 17;10(3):e0118174. doi: 10.1371/journal.pone.0118174 (PMC4363688; doi:10.1371/journal.pone.0118174)
Supplement: S1 Protocol — (DOCX) [file pone.0118174.s002.docx]

**STUDY PROTOCOL**

| **Official Title** | **EFFECT OF PARICALCITOL vs CALCITRIOL ON HEMOGLOBIN LEVELS IN CKD PATIENTS: A RANDOMIZED TRIAL** |
| --- | --- |
| **Short Title** | Paricalcitol effect on anemia in CKD |
| **Start Date** | October 2010 |
| **Brief Summary** | Current activated Vitamin D therapies are approved for treating secondary hyperparathyroidism in chronic kidney disease (CKD), and a large body of experimental data in animals confirms the effects of Vitamin D that extend beyond mineral metabolism. Several studies show that the benefits are greater with the newer vitamin D analog paricalcitol when compared with calcitriol. A large gap exists in our knowledge between epidemiological studies in human that demonstrate improved outcomes with vitamin D use and observations in preclinical studies demonstrating the pleiotropic effects of Vitamin D. To explore the provenance of epidemiological outcomes in CKD, we conducted a pilot randomized trial to determine whether the use of paricalcitol, compared to calcitriol, leads to improvement in anemia, a marker associated with worse outcomes in chronic kidney disease, and whether this effect not only reflects the hyperparathyroidism correction, but is also dependednt on the direct effects of paricalcitol on erythroid progenitor cells.  To better understand the direct effects of paricalcitol on anemia in patients with chronic kidney disease (stage 3-5), we conducted a pilot trial on 60 patients who were randomly assigned to receive calcitrol or paricalcitol for 6 months. |
| **Study Design** | Intervention Model: Parallel Assignment Number of arms: 2  Masking: Open Label |
| **Condition** | Anemia  Chronic kidney disease |
| **Intervention** | Drug: Paricalcitol  Other Name: Zemplar.  Drug: Calcitriol  Other name: Rocaltrol |
| **Study Arm** | Two arms:  Active comparator: Calcitriol.  The calcitriol dosage schedule provided for an initial dose of 0.5 mcg every other day and titration was performed on the basis of serum levels of intact PTH (iPTH), Ca. P, and Ca x P product as suggested by the US National Kidney Foundation Dialysis outcomes Quality Initiative (NKF-DOQI) and Kidney disease: Improving Global Outcomes (KDIGO) guidelines.  Experimental: Paricalcitol  The paricalcitol initial dose was 1 mcg/day and titration was performed on the basis of the levels of iPTH, Ca, P, and Ca x P product as suggested by the NKF-DOQI and KDIGO guidelines. |
| **Eligibility Criteria** | Inclusion criteria were: age <18 years; written informed consent; CKD stage 3-5 (eGFR <60 ml/min), Hb levels between 10 and 12.5 g/dL, normal transferrin saturation (TSAT, 20-40%), plasma ferritin levels ≥100 ng/mL, normal mean corpuscular volume (MCV, 85-95 fL), parathormone (PTH) serum levels between 20 and 300 pg/mL, according to the suggested values for kidney disease stage, and calcium and phosphate plasma levels within their normal values (i.e. <10.5 mg/dl, and <4.5 mg/dl, respectively), controlled by a low protein diet (0.7-0.9 g/kg b.w./day), calcium supplements and phosphate binders.  Exclusion criteria were: presence of inflammatory, infectious disease or surgical interventions in the last 3 months, high-sensitivity C-reactive protein (CRP) levels >3 mg/dl (normal values: 0-5 mg/ml), hematological disorders or bleeding in the last 6 months, malignancies, treatment with immunosuppressive drugs, poorly controlled hypertension (>170/100 mmHg), presence of clinical/ECG signs of cardiovascular disease in the last 3 months. |
| **Gender** | Both males and females |
| **Ages** | From 18 Years |
| **Accepts Healthy Volunteers** | No |
| **Primary Endpoints** | The primary endpoint of the study was to evaluate the effects of low doses of two different treatments, namely calcitriol and paricalcitol, on Hb plasma levels during a follow-up period of 6 months in CKD patients with stable levels of calcium, phosphorus and PTH. As secondary end point, the modifications in UProt with both drugs were also evaluated. |
| **Primary Endpoint Measure** | - Modification in hemoglobin levels   [Time Frame: 6 months] [ Designated as safety issue: No ] |
| **Secondary Endpoints** | - As secondary end point, the modifications in urinary protein excretion with both drugs were evaluated. |
| **Secondary Endpoint Measure** | - Modification in urinary protein levels - [Time Frame: 6 months] [ Designated as safety issue: No ] |
